# Retraction: A hybrid upconversion nanoprobe for ratiometric detection of aliphatic biogenic amines in aqueous medium

**DOI:** 10.1039/d5na90080h

**Published:** 2025-11-04

**Authors:** Shilpi Jaiswal, Subhankar Kundu, Sujoy Bandyopadhyay, Abhijit Patra

**Affiliations:** a Department of Chemistry, Indian Institute of Science Education and Research Bhopal Bypass Road, Bhauri Bhopal 462066 Madhya Pradesh India abhijit@iiserb.ac.in

## Abstract

Retraction of ‘A hybrid upconversion nanoprobe for ratiometric detection of aliphatic biogenic amines in aqueous medium’ by Shilpi Jaiswal *et al.*, *Nanoscale Adv.*, 2021, **3**, 3232–3239, https://doi.org/10.1039/D0NA00995D.

The Royal Society of Chemistry, with the agreement of the named authors, hereby wholly retracts this *Nanoscale Advances* article due to concerns with the reliability of the data.

Concerns were raised over the inset of Fig. 4c, 7d, e and S16. In the inset of Fig. 4c, the gold and teal lines have the same noise pattern. In Fig. 7, sub-images d and e are the same photos. In Fig. S16 there are repetitions of the decay curves, specifically in (a) curve HN_4_ and (b) curves 2.0 and 4.5 spermine. The authors were contacted for comment and admitted to the errors in multiple figures in the article and provided a new set of data for Fig. 4c, original images for Fig. 7d, e, and raw and reanalysed data for Fig. S16 for correction.

An independent expert assessed the responses and data supplied by the authors but has found this does not correspond consistently to the published results. Following the advice of the independent expert, the authors have agreed to the retraction of this paper and apologised for these issues. The editors consider the results and conclusions invalid.

The corresponding author has provided this contribution statement below.

S. J. and S. K. conceptualized the work upon discussing with A. P.; S. J. acquired and analyzed most of the data for this manuscript and wrote the original draft of the paper with assistance from S. K.; S. B. initiated the synthesis of the TDPM molecule. A. P. supervised the project and corrected the manuscript.

The authors were informed about the retraction of the article. S. K., S. B. and A. P. have agreed with the decision; S. J. has not responded.

Signed: Subhankar Kundu, Sujoy Bandyopadhyay and Abhijit Patra, 28th October 2025.

Retraction endorsed by Jeremy Allen, Executive Editor, *Nanoscale Advances*.

